# Diagnosis and Treatment of Multiseptate Gallbladder with Recurrent Abdominal Pain

**DOI:** 10.1155/2011/162853

**Published:** 2011-10-19

**Authors:** Turgut Karaca, Omer Yoldas, Bulent Caglar Bilgin, Selma Bilgin, Ender Evcik, Saadet Ozen

**Affiliations:** ^1^Department of General Surgery, Ankara Occupational Diseases Hospital, Ankara, Turkey; ^2^Department of General Surgery, Ordu State Hospital, Ordu 52200, Turkey; ^3^Department of Anatomy, Faculty of Medicine Hacettepe University, Ankara, Turkey; ^4^Department of Radiology, Ankara Occupational Diseases Hospital, Ankara, Turkey

## Abstract

Laparoscopic cholecystectomy is usually performed for gallstones or polyp of the gallbladder. Multiseptate gallbladder is a rare congenital malformation. Although several asymptomatic cases have been described, patient usually present with right upper abdominal pain. We present a 29-year-old female patient with multiseptate gallbladder, cholecystectomy was performed, and her abdominal pain and gastrointestinal complaints have resolved.

## 1. Introduction 

Multiseptate gallbladder (MSG) is a rare congenital malformation of the gallbladder. Patients with MSG usually admits to the emergency services with differential symptoms such as right upper abdominal pain, nause and vomiting, and abdominal complaints. We present a case of MSG with recurrent abdominal pain in which laparoscopic cholecystectomy resolves the complaints of the patient. 

## 2. Case Report

**Figure 1:**
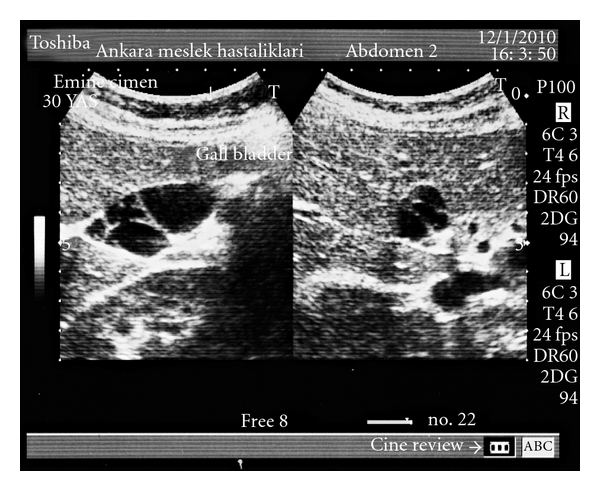
Multiple linear echoes mimicking honey-comb pattern.

**Figure 2:**
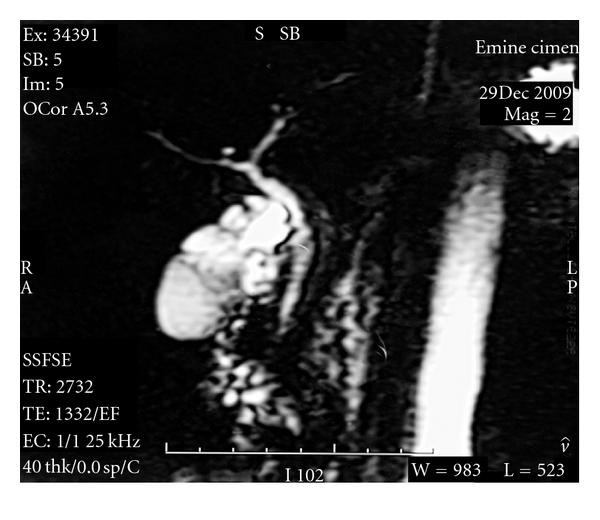
Grapelike cluster of the gallbladder in MRCP.

**Figure 3:**
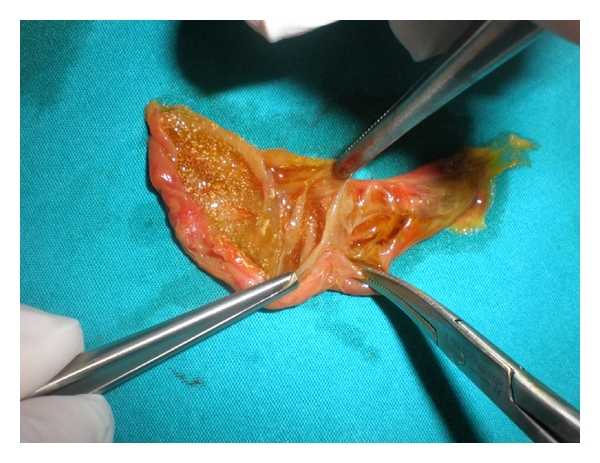
Macroscopic examination demonstrates multiple edematous septa dividing the gallbladder lumen into compartments.

 A 29-year-old female patient was admitted to our hospital with a complaint of recurrent right upper quadrant pain. The pain was associated with nause and dyspepsia especially after fatty foods. Physical examination and laboratory tests (CBC, liver function tests, serum amylase, gamma glutamyl transpeptidase, and alkaline phosphatase levels) revealed no abnormality. An abdominal ultrasound was obtained with the suspect of gallstone. There were multiple linear echoes within the gallbladder dividing the lumen into compartments mimicking a honey-comb pattern (Figure 1). The wall thickness of the gallbladder was normal, and there were no gallstones. Magnetic resonance cholangiopancreatography (MRCP) was performed and showed a grapelike cluster of the gallbladder (Figure 2). According to these findings, multiseptate gallbladder was diagnosed. Laparoscopic cholecystectomy was performed and macroscopic examination demonstrated multiple edematous septa dividing the gallbladder lumen into compartments (Figure 3). The patient was discharged on postoperative first day without complication and followed up for a six-month period. Her abdominal pain and gastrointestinal complaints have resolved after the surgery. 

## 3. Discussion 

Multiseptate gallbladder (MSG) is a rare congenital malformation of the gallbladder. It most likely results from incomplete vacuolization of the developing gallbladder bud or persistent “wrinkling” of the gallbladder wall [1, 2]. There are multiple septa which give a honeycomb appearance in MSG, and these septa usually involve the lumen of the entire gallbladder whereas they are sometimes found in only a portion of the gallbladder [3–6]. The causes of multiseptate appearance of the gallbladder on sonography include gallbladder diseases such as multiseptate gallbladder, hyperplastic cholecystosis, and cholecystitis [7]. Asymptomatic patients are very rare in literature [3], most patients present with long term abdominal symptoms such as right upper quadrant tenderness, recurrent abdominal pain, nause and vomiting, and gastrointestinal complaints. Septa are the reason of impaired motility of the gallbladder, and this causes a stasis in the bile flow, and it seems to be the reason of recurrent abdominal pain. Ultrasound (US) evaluation of the gallbladder is usually sufficient to diagnose MSG although other modalities such as computed tomography, magnetic resonance cholangiopancreatography (MRCP) and endoscopic retrograde cholangiography (ERCP) have been described to establish the diagnosis. Ultrasound appearances consist of multiple linear echoes that can be seen to cross the gallbladder lumen, producing a honey-comb appearance [8]. The combination of US and MRCP is the most useful and the least invasive methods to diagnose multiseptate gallbladder. 

## 4. Conclusion

In conclusion, multiseptate gallbladder is a rare congenital anomaly and a rare cause of recurrent abdominal pain. Cholecystectomy is the choice of treatment in symptomatic patients such in our case. Our patients' abdominal pain and gastrointestinal complaints have resolved after surgery. Cholecystectomy should also be considered in elderly, asymptomatic patients in whom MSG is incidentally discovered, due to the possibility of undetected carcinoma of the gallbladder [9].
